# A novel approach using the five-flap technique for posterior fourchette reconstruction: a clinical study addressing flap deformity and sexual dysfunction

**DOI:** 10.3389/fsurg.2025.1571769

**Published:** 2025-05-09

**Authors:** Yilin Li, Meichen Liu, Yu Zhou, Yujiao Cao, Fengyong Li

**Affiliations:** Urogenital Reconstructive & Gender-affirming Center, Plastic Surgery Hospital, Chinese Academy of Medical Sciences and Peking Union Medical College, Beijing, China

**Keywords:** posterior fourchette, five-flap technique, sexual function improvement, vulvar aesthetic reconstruction, flap deformities

## Abstract

**Objective:**

To evaluate the clinical efficacy of the five-flap technique in the reconstruction of posterior fourchette flap deformities.

**Methods:**

A retrospective analysis was conducted on 36 patients with posterior fourchette flap deformities who underwent surgery using the five-flap technique. Preoperative and postoperative pain scores, sexual function scores, and vulvar aesthetic scores were recorded. Additionally, the degree of sexual function improvement and the incidence of complications were assessed. Depending on the distribution of the data, paired *t*-tests or non-parametric tests were employed to compare preoperative and postoperative scores. Descriptive statistics summarized the distribution of sexual function improvements and complications.

**Results:**

The study demonstrated a significant reduction in postoperative pain scores [preoperative: 1.5 [0, 2] vs. postoperative: 0 [0, 1], *p* = 0.001]. Sexual function scores improved from 19.939 ± 3.903 to 28.742 ± 2.766 (*p* = 0.019), while aesthetic scores increased from 10.50 (9, 12.75) to 21.72 (20.25, 23) (*p* < 0.001). Based on the definition of sexual function improvement (significant improvement, moderate improvement, no improvement), 72.2% of patients experienced significant improvement, 25.0% moderate improvement, and only 2.8% showed no improvement. Furthermore, no cases of flap necrosis or recurrence were observed. The complication rate was low, with only one case (2.8%) of mild infection and two cases (5.6%) of scar tension, all of which resolved well with local treatment.

**Conclusion:**

The five-flap technique is a safe and effective innovative reconstructive method that demonstrates significant pain relief, sexual function improvement, and aesthetic enhancement in the treatment of posterior fourchette flap deformities. With a low complication rate, it provides a personalized treatment option for complex vulvar deformities.

## 1 Introduction

The posterior fourchette, located at the rear of the female vulva, connects the posterior ends of the labia minora and plays a crucial role in maintaining the normal anatomical structure and function of the vulva (1, 2). However, factors such as congenital developmental abnormalities, trauma, or surgical interventions may damage the posterior fourchette, resulting in flap deformities that affect both the aesthetics and functionality of the vulva, potentially leading to sexual dysfunction (3–5). Flap deformities not only negatively impact patients' psychological well-being but may also cause painful sexual intercourse, increased risk of infections, and other physiological complications (6). Traditional repair methods, such as simple excision and suturing, can temporarily address these issues in some cases. However, these approaches often lack a targeted design, leading to increased local tension, scar contracture, and suboptimal postoperative functional recovery (7, 8). In recent years, advancements in plastic surgery techniques have led to the emergence of procedures aimed at both functional and aesthetic vulvar reconstruction. For instance, Dong Yulin et al. proposed the labial margin arc-shaped excision combined with crescent-shaped excision technique to treat labial hypertrophy with excessive clitoral hood, yielding excellent clinical outcomes (9). Similarly, Zheng Meilian et al. employed arc-shaped excision of the elongated clitoral hood alongside linear labial margin excision, achieving satisfactory postoperative aesthetics, fewer complications, and reduced surgical difficulty (10). These approaches have significantly improved patient satisfaction and quality of life.

However, there is still a lack of standardized and effective techniques specifically designed for the repair of posterior fourchette flap deformities. Existing literature primarily focuses on the reconstruction of the labia minora or other parts of the vulva, with limited research addressing the unique anatomical and functional characteristics of the posterior fourchette (11). Therefore, developing a repair technique that not only restores the normal anatomical structure but also enhances functionality is critical for addressing posterior fourchette flap deformities and their associated dysfunctions.

This study aims to propose and validate an innovative repair method based on the five-flap technique to tackle the challenges of posterior fourchette flap deformity repair. Addressing the limitations of existing methods, this study systematically evaluates the clinical value of the five-flap technique in terms of anatomical reconstruction, aesthetic improvement, and functional recovery through clinical observation and follow-up.

## 2 Materials and methods

### 2.1 Patient selection

This retrospective study analyzed patients who underwent repair of posterior fourchette flap deformities using the five-flap technique at Plastic Surgery Hospital, Chinese Academy of Medical Sciences and Peking Union Medical College between January 2022 and March 2024. Based on the following inclusion and exclusion criteria, 36 patients were ultimately included. Postoperative follow-up lasted for three months, with data collected via telephone or outpatient visits.

### 2.2 Inclusion criteria

1. Patients diagnosed with posterior fourchette flap deformities by professional gynecologists or plastic surgeons.
2. Patients who underwent surgical repair using the five-flap technique.
3. Aged 18–45 years, without severe systemic comorbidities.
4. Complete medical records, including preoperative diagnosis, intraoperative details, and postoperative follow-up data.
5. Signed informed consent agreeing to participate in this study.

### 2.3 Exclusion criteria

1. Patients with incomplete case records or missing follow-up data.
2. Patients with severe infections, autoimmune diseases, or other systemic illnesses that could affect surgical outcomes.
3. Patients who had undergone prior surgeries in the posterior fourchette region that might influence the results. This included but was not limited to: previous posterior fourchette repair, perineoplasty, posterior vaginal wall repair, episiotomy repair with complications, and vulvoperineal reconstruction following trauma or obstetric injury.
4. Patients with follow-up periods of less than 3 months or who were lost to follow-up.

All patients met the same inclusion and exclusion criteria. This study was approved by the Ethics Committee of Plastic Surgery Hospital, Chinese Academy of Medical Sciences and Peking Union Medical College.

### 2.4 Outcome measures

All patients were evaluated preoperatively and at 3 months postoperatively using the following standardized assessment tools:

1. Pain Assessment: Pain scores were assessed using the Visual Analogue Scale (VAS), a validated tool ranging from 0 (no pain) to 10 (worst imaginable pain). Patients were specifically asked to rate pain experienced during or immediately after sexual intercourse.
2. Sexual Function Assessment: Sexual function was evaluated using the Female Sexual Function Index (FSFI), a 19-item questionnaire assessing six domains of sexual function: desire, arousal, lubrication, orgasm, satisfaction, and pain. Total scores range from 2 to 36, with higher scores indicating better sexual function.
3. Aesthetic Assessment: Vulvar aesthetics were evaluated using a modified five-point Likert scale questionnaire designed specifically for this study. The questionnaire included five aspects of vulvar appearance: symmetry, scarring, natural contour, tissue tension, and overall appearance, with scores ranging from 4 to 25. Higher scores indicate greater aesthetic satisfaction.
4. Sexual Function Improvement Categories: The degree of sexual function improvement after surgery was categorized as follows:
  • Significant improvement: defined as a postoperative FSFI score increase of ≥6 points compared to the preoperative score or a postoperative score of 30 points or higher.
  • Moderate improvement: defined as a postoperative FSFI score increase of 3–5 points or a postoperative score between 26 and 29 points.
  • No improvement: defined as a postoperative FSFI score increase of less than 3 points or a score still below 26 points.

These categories were established based on both quantitative score changes and qualitative patient feedback during follow-up consultations.

### 2.5 Surgical procedure

**Figure 1:**
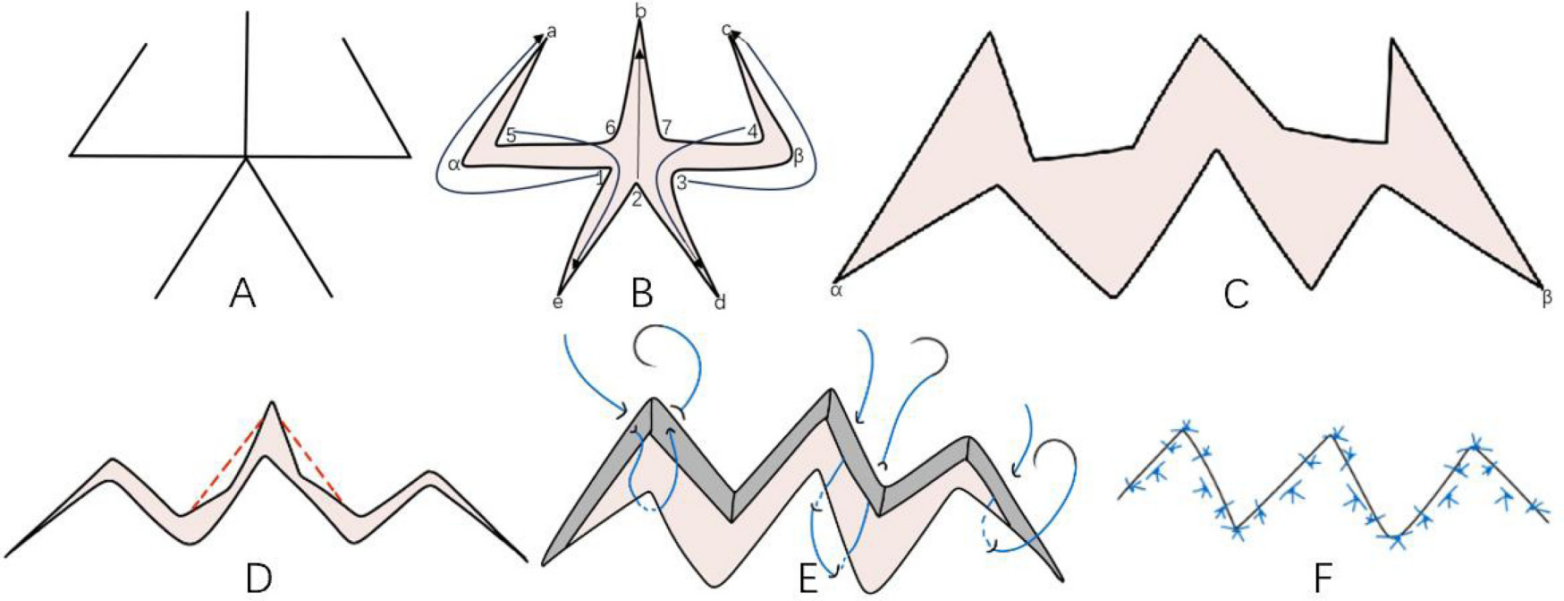
Surgical design diagram; **(A)** five-flap desgin; **(B)** flap transpostion layout; **(C)** post-transposition flap effect; **(D)** trim along the red line to smoth edges; **(E)** preliminary suture desgin; **(F)** final suture outcome.

Preoperatively, a five-flap incision design was planned based on the specific characteristics of the patient's posterior fourchette flap deformity. The five-flap method combines two “Z-plasty” and one “Y-V plasty” techniques, designed with the primar*y* axis along the highest tension line of the flap deformity. At each end of the primar*y* axis, two arms were extended laterally to a length equal to half the primar*y* axis, forming angles of 60°–65° with the axis. Perpendicular to the midpoint of the axis on one side, a line slightly shorter than the arm length was drawn. On the opposite side of the midpoint, two diagonal arms, each equal in length to half the primar*y* axis, were drawn to form three 60° angles with the axis (Figure 1A).

During surgery, incisions were made along the design lines through the skin and subcutaneous tissue, forming five flaps. Subsequently, according to the plan, angles 1–5 are sequentially transferred to target positions A–E (Figure 1B), creating the structure shown in Figure 1C. During the transfer process, Flaps 6 and 7 might stretch into near-180° obtuse angles; to ensure a smooth incision edge, these areas were trimmed along the red lines as necessary (Figure 1D). Three suturing techniques were employed: (i) At the sharp corners, sutures were placed by inserting the needle into the skin at the incision edge, passing through the subcutaneous tissue at the corner, and exiting to tie a knot. (ii) Parallel mattress sutures were used for the middle portion of each edge. (iii) Interrupted sutures were added to the ends of the edges as needed (Figure 1E). The final outcome formed a zigzag suture line (Figure 1F), effectively alleviating contracture and restoring the anatomical structure and function by extending tissue length and optimizing tension distribution. The surgical wound was covered with sterile dressings, and flap color and hematoma formation were closely monitored postoperatively.

### 2.6 Statistical analysis

All statistical analyses were performed using SPSS 25.0 (SPSS Inc.). Continuous variables following a normal distribution were expressed as mean ± standard deviation and assessed using the *t*-test. Variables not following a normal distribution were expressed as median (interquartile range) and evaluated using the nonparametric rank-sum test for group comparisons. A *P*-value < 0.05 was considered statistically significant.

## 3 Results

A total of 36 patients were included in this study, with ages ranging from 18 to 45 years, and an average age of 30.39 ± 5.97 years.

### 3.1 Comparison of functional scores preoperatively and at 3 months postoperatively

The median preoperative pain score was 1.5 (interquartile range: 0, 2), which significantly decreased to 0 (interquartile range: 0, 1) at 3 months postoperatively. Pain scores were assessed using the Visual Analogue Scale (VAS) as described in the Methods section. The Wilcoxon signed-rank test showed that postoperative pain scores were significantly lower than preoperative scores (*p* = 0.001), demonstrating the surgery's effectiveness in alleviating pain. The mean preoperative sexual function score (20.583 ± 4.892), assessed using the Female Sexual Function Index (FSFI) as detailed in the Methods section, increased to 28.497 ± 2.916 at 3 months postoperatively. A paired *t*-test showed that postoperative sexual function scores were significantly higher than preoperative scores (*p* = 0.011), indicating a significant improvement in sexual function. The median preoperative aesthetic score was 10.50 (interquartile range: 9, 12.75), which significantly increased to 21.72 (interquartile range: 20.25, 23) at 3 months postoperatively. Aesthetic scores were assessed using a modified five-point Likert scale as described in the Methods section, ranging from “completely dissatisfied” to “very satisfied” regarding vulvar appearance. The Wilcoxon signed-rank test showed that postoperative scores were significantly higher than preoperative scores (*p* < 0.001), indicating a significant improvement in aesthetics (Table 1).

**Table 1:** Comparison of functional scores preoperatively and at 3 months postoperatively.

Variable	Preoperative	3 months postoperative	Test statistic	*P*-value
Pain score	1.5 (0, 2)	0 (0, 1)	Z^a^ = 3.309	0.001
Sexual function Score	19.939 ± 3.903	28.742 ± 2.766	*t*^b^ = 11.041	0.019
Aesthetic score	10.50 (9, 12.75)	21.72 (20.25, 23)	Z^a^ = 7.283	<0.001

^a^ Independent samples *t*-test.

^b^ Wilcoxon nonparametric rank-sum test.

### 3.2 Sexual function improvement

The degree of sexual function improvement after surgery was divided into three categories as described in the Methods section. The results showed that 26 patients (72.2%) had significant improvement, 9 patients (25.0%) had moderate improvement, and 1 patient (2.8%) had no improvement (Table 2).

**Table 2:** Statistics on sexual function improvement after five-flap technique surgery.

Degree of sexual function improvement	Cases (*n*)	Percentage (%)
Significant improvement	26	72.2%
Moderate improvement	9	25.0%
No improvement	1	2.8%

### 3.3 Postoperative indicators

During the postoperative follow-up period, 1 patient (2.8%) experienced mild infection, and 2 patients (5.6%) developed scar tension issues, all of which resolved with local treatment and without severe complications. At 10 days post-surgery, healing assessments showed that 34 patients (94.4%) had good healing, 2 patients (5.6%) had moderate healing, and none had poor healing outcomes. At 3 months post-surgery, none of the 36 patients experienced recurrence or flap necrosis (Table 3).

**Table 3:** Statistical results of postoperative related indicators.

Postoperative related indicators	Cases (*n*)	Percentage (%)
Infection	1	2.8%
Scar tension	2	5.6%
Good wound healing	34	94.4%%
Moderate wound healing	2	5.6%
Poor wound healing	0	0%
Recurrence	0	0%
Flap necrosis	0	0%

### 3.4 Typical case

**Figure 2:**
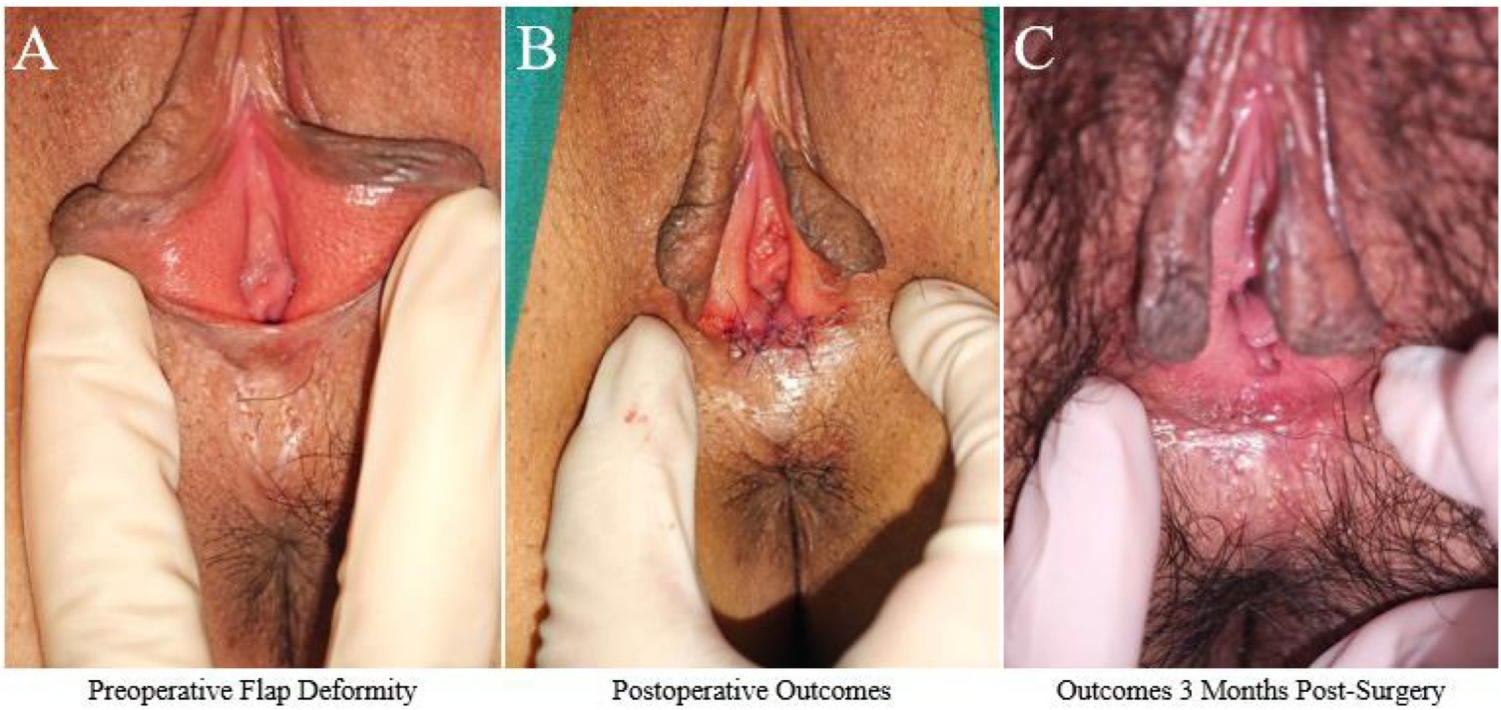
Typical case 1. **(A)** Preoperative flap deformity: Small fissure visible at the midline of the posterior fourchette with flap-like elevation on bilateral traction. **(B)** Postoperative outcomes: Immediate result after posterior fourchette reconstruction using the five-flap technique. **(C)** Outcomes 3 months post-surgery: Complete resolution of sexual pain with natural restoration of vulvar appearance.

Case 1. A 32-year-old female was admitted with a chief complaint of “pain in the posterior fourchette after sexual intercourse for 2 years.” Past medical history: no significant medical history, no history of childbirth or abortion. Physical examination showed a well-developed vulva, with a small fissure visible at the midline of the posterior fourchette. Bilateral traction revealed flap-like elevation (Figure 2A). Preoperative routine blood tests, coagulation function tests, and infectious disease screening showed no abnormalities. The patient underwent posterior fourchette reconstruction using the five-flap technique under local anesthesia in the outpatient operating room. The incision design was precise, and the reconstruction was performed using 6-0 absorbable sutures (Figure 2B). The procedure was uneventful. The patient was observed in the hospital for 30 min postoperatively and discharged without complications. She was instructed to perform a sitz bath with potassium permanganate solution twice daily for 15 min each time. At 10 days post-surgery, a follow-up examination showed that the incision was healing well, with normal flap vascularization and no signs of infection or scar tension. The patient avoided strenuous activities for 1 month postoperatively and gradually resumed sexual activity two months after surgery. At the 3-month follow-up, the patient reported complete resolution of sexual pain, significant improvement in sexual satisfaction, and natural restoration of vulvar appearance (Figure 2C). The patient expressed high satisfaction with the surgical outcome.

**Figure 3:**
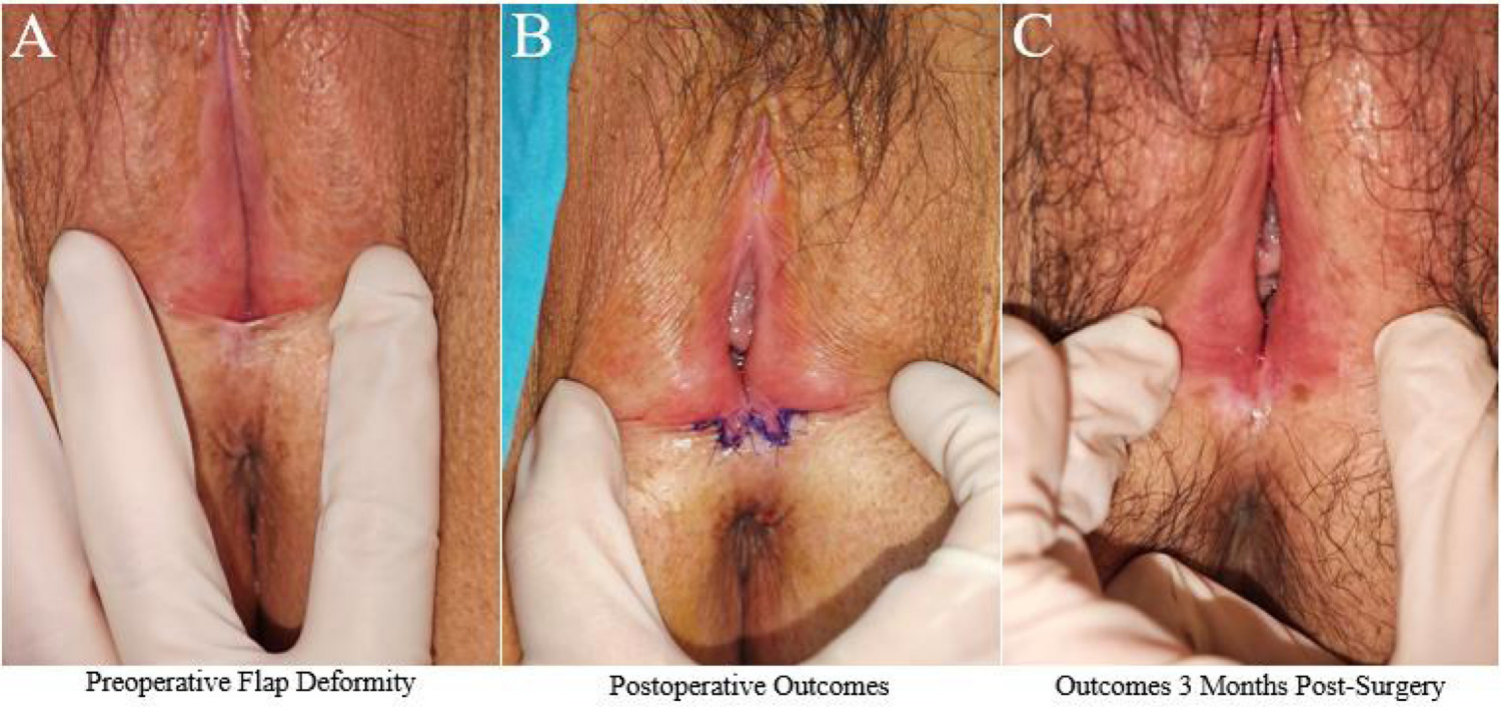
Typical case 2. **(A)** Preoperative flap deformity: Lichen sclerosus changes in the vulva with localized labial atrophy and posterior fourchette adhesion exhibiting flap-like elevation upon traction. **(B)** Postoperative outcomes: Immediate result after reconstruction using the five-flap technique. **(C)** Outcomes 3 months post-surgery: Normal sexual activity restored with natural posterior fourchette morphology and alleviation of sexual pain.

Case 2. A 40-year-old female was admitted with a chief complaint of “vulvar lichen sclerosus for 4 years, pain and tearing at the posterior fourchette during intercourse, and inability to engage in normal sexual activity for 1 year.” The patient was diagnosed with vulvar lichen sclerosus 4 years ago and had undergone conservative treatments such as medication and laser therapy with poor results. She had no history of childbirth or abortion. Physical examination revealed lichen sclerosus changes in the vulva, localized labial atrophy of the labia minora, and adhesion of the posterior fourchette, which exhibited flap-like elevation upon traction (Figure 3A). Preoperative routine blood tests, coagulation function tests, and infectious disease screening showed no abnormalities. The patient underwent posterior fourchette reconstruction using the five-flap technique under local anesthesia in the outpatient operating room. The procedure was performed using 6-0 absorbable sutures (Figure 3B) and was uneventful. The patient was observed in the hospital for 30 min postoperatively and discharged without discomfort. She was instructed to perform a sitz bath with potassium permanganate solution twice daily for 15 min each time. At 10 days post-surgery, a follow-up examination showed good incision healing and normal flap vascularization. The patient avoided strenuous activities for 1 month postoperatively and gradually resumed sexual activity 2 months after surgery. At the 3-month follow-up, the patient reported a return to normal sexual activity, natural restoration of the posterior fourchette morphology (Figure 3C), alleviation of sexual pain, and significant improvement in satisfaction.

## 4 Discussion

posterior fourchette flap deformity is a relatively rare anatomical abnormality that significantly impacts both the physiological function and psychological well-being of patients (3). Due to the complex anatomical structure of this region, which serves as an important barrier and support while directly participating in sexual and reproductive functions, deformities often result in dyspareunia, tissue tearing, and significant vulvar aesthetic abnormalities, severely affecting the patient's quality of life (12, 13). However, current research on reconstruction techniques for deformities in the posterior fourchette region is limited, with most studies focusing on functional and aesthetic repair of other areas, such as the labia minora or clitoris (14–19). Although existing methods have achieved some progress in localized improvements, their effectiveness in addressing the complex deformities of the Posterior fourchette is often limited due to the unique anatomical characteristics and functional demands of this region. These limitations highlight the need for further exploration in this field. Based on the five-flap technique, this study proposes an innovative repair method to address this technical gap.

As a flexible multi-flap incision repair method, the five-flap technique has provided crucial theoretical support and practical insights for this study through its successful application in various fields of plastic surgery in recent years. For instance, the five-flap technique has been widely used in the correction of axillary webbed scar contractures, thumb web space contractures, and syndactyly deformities (20, 21). Studies have shown that by redistributing tension and optimizing flap cross-positioning, this technique not only restores local function but also enhances postoperative aesthetic outcomes, significantly improving patient satisfaction (22, 23). Additionally, the application of the five-flap method in minor cup ear correction and epicanthoplasty has further validated its advantages in addressing complex, small-scale tissue deformities (24–26). These studies demonstrate the broad applicability and favorable clinical outcomes of the five-flap technique in repairing complex deformities.

Similarly, this study is the first to apply the five-flap technique to the reconstruction of posterior fourchette flap deformities. Through multi-flap incision design, the technique achieves tension redistribution, reduces the risk of postoperative scar contracture and functional recurrence, and significantly improves postoperative aesthetic outcomes. Postoperative follow-up data revealed that pain scores significantly decreased [1.5 [0, 2] vs. 0 [0, 1], *p* = 0.001], while sexual function scores (19.939 ± 3.903 vs. 28.742 ± 2.766, *p* = 0.019) and vulvar aesthetic scores [10.50 [9, 12.75] vs. 21.72 [20.25, 23], *p* < 0.001] showed remarkable improvement. These findings align with the successful application of the five-flap technique in other fields, further validating its feasibility in the reconstruction of complex vulvar deformities.

Compared to traditional excision and suturing methods, the five-flap technique demonstrated higher safety in this study. None of the patients experienced flap necrosis or recurrence, and the complication rate was low, with only 1 patient (2.8%) reporting mild infection and 2 patients (5.6%) reporting postoperative scar tension issues. These issues were effectively resolved through local treatment and did not significantly impact the overall recovery of the patients. Notably, this study highlighted an important advantage over the approach by Yuanbo et al. (27), which involved pulling the mucosal flaps outward. In contrast, our method repositions the skin tissue inward, ensuring that the postoperative surface exposed to friction is primarily the skin. This adjustment enhances durability and flexibility, significantly reducing the risk of recurrent tears or injuries. Overall, the five-flap technique showed comprehensive advantages in alleviating postoperative pain, optimizing anatomical function, and improving aesthetic outcomes, providing a reliable technical option for the repair of complex vulvar deformities.

Although this study confirmed the effectiveness of the five-flap technique in posterior fourchette deformity repair, some limitations exist. First, the sample size was relatively small (36 cases), which may limit the generalizability of the results. Second, the follow-up duration was short (3 months), which did not allow for a full evaluation of the long-term outcomes, such as sustained functional recovery and patient satisfaction. Additionally, this study was based on cases from a single center, which may introduce selection bias. Future research should aim to include larger sample sizes, multicenter cases, and extended follow-up durations to further validate the long-term safety and efficacy of the five-flap technique.

## 5 Conclusion

In summary, this study validated the safety and efficacy of the five-flap technique in the repair of posterior fourchette flap deformities. This technique significantly alleviates postoperative pain and improves patients' sexual function and vulvar aesthetic scores without increasing the risk of complications. By utilizing a multi-flap incision design, the technique effectively redistributes tension, achieving the dual goals of anatomical function restoration and aesthetic improvement. Compared to traditional excision and suturing methods, the five-flap technique demonstrates significant advantages in the reconstruction of complex vulvar deformities, offering patients a safe and personalized treatment option. The application of this innovative technique fills a technical gap in vulvar plastic surgery and provides new directions for future research.

## Data Availability

The original contributions presented in the study are included in the article/Supplementary Material, further inquiries can be directed to the corresponding author.
